# Ionization Study of Isomeric Molecules in Strong-field Laser Pulses

**DOI:** 10.1038/srep42149

**Published:** 2017-02-10

**Authors:** Stefan Zigo, Anh-Thu Le, Pratap Timilsina, Carlos A. Trallero-Herrero

**Affiliations:** 1 J.R. Macdonald Laboratory, Department of Physics, Kansas State University, Manhattan, Kansas 66506, USA

## Abstract

Through the use of the technique of time-of-flight mass spectroscopy, we obtain strong-field ionization yields for randomly oriented 1,2-dichloroethylene (1,2-DCE) (C_2_H_2_Cl_2_) and 2-butene (C_4_H_8_). We are interested in studying the effect of conformal structure in strong-field ionization and, in particular, the role of molecular polarity. That is, we can perform strong-field ionization studies in polar vs non-polar molecules that have the same chemical composition. We report our findings through the ionization yields and the ratio (trans/cis) of each stereoisomer pair as a function of intensity.

Strong-field ionization of atoms and molecules occurs when one or more electrons are stripped from the parent molecule by an electric field of a relatively low frequency^1^. Unlike photoionization by single photons where the field can be very weak, in the strong-field limit, the binding potential of the electron is distorted by the field. This difference poses the question of the sensitivity of strong-field ionization to the molecular structure. While the sensitivity of strong-field ionization to molecular orbitals has been demonstrated^2^,^3^,^4^, the limits on sensitivity to molecular structure have not been thoroughly studied yet. To partially answer this question, many studies have been done in the past looking at ionization ratios between atoms and molecules^5^,^6^,^7^,^8^. From the theoretical point of view, target structure enters by virtue of the different quantum states that the electron has access to or the possible quantum paths en route to ionization^1^,^9^,^10^,^11^,^12^,^13^,^14^,^15^,^16^. In the limit where many photons are absorbed, the number of quantum paths is very large, and ionization can more adequately be described through tunneling across the finite barrier created by the strong field. To differentiate all the possible regimes, we follow the definitions first introduced by Keldysh^1^. The three different regimes are: the multi-photon (MPI) regime, where ionization occurs at low intensities, the tunneling (TI) regime, where laser intensities are strong enough to deform the potential of the atom or molecule such that quantum tunneling can occur, and the over-the-barrier (OBI) regime where the intensity is so high that the deformed potential falls below the energy level of the electron allowing ionization to occur. The so-called Keldysh parameter, 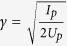, where *I*_*p*_ is the ionization potential and *U*_*p*_ is the ponderomotive potential, is one parameter that allows one to establish the different regimes. For 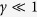, ionization occurs through tunneling and for 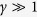, ionization of an electron has more distinguishable quantum paths and is called the multiphoton regime.

In this report, we look at the ionization yields of the first ion of the isomeric molecules *cis*- and *trans*-1,2-dichloroethylene (1,2-DCE) and *cis*- and *trans*-2-butene (Fig. 5(b–e)). Both samples were purchased from Sigma Aldrich with the following purification: 97% *cis*-1,2-DCE, 98% *trans*-1,2-DCE, and 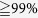 *cis*- and *trans*-2-butene. These molecules have ionization potentials of 9.11 ± 0.01 eV and 9.10 ± 0.01 eV for *cis*- and *trans*-2-butene and 9.66 ± 0.01 eV and 9.64 ± 0.02 eV for *cis*- and *trans*-1,2-DCE respectively^17^. Each isomer is studied separately under identical conditions. Please see the **Experimental Results** and **Methods: Experimental Setup** sections for further details.

To date, there have been many studies on atomic and molecular ionization^5^,^6^,^7^,^18^,^19^, however, there are only a few that investigate the difference in ionization between polar and non-polar molecules^20^. The stereoisomers studied in this report provide an excellent way to investigate the effects of polarity on strong-field ionization yields since the *cis*-isomers are polar and the *trans*-isomers are non-polar. The calculated dipole moment of the highest occupied molecular orbital (HOMO) of *cis*-1,2-DCE is -0.75 au and *cis*-2-butene is -0.97 au. Please see the **Theoretical Results** section for further details.

In this paper, we show that experimentally, for randomly oriented isomeric molecules, polarity does not strongly influence ionization yield. However, we find large differences in the ionization yield between isomers that cannot be explained by the current theories. We check this with three different modern methods for finding molecular ionization rates, strong-field approximation (SFA)^1^,^21^, molecular orbital PPT (MO-PPT) theory^9^,^22^, and molecular orbital ADK theory (MO-ADK)^16^. All of which stem from the Keldysh theory of ionization, but utilize the more correct inclusion of the Coulomb interaction on the final electron state which Keldysh neglected.

To further motivate this study, we should mention that recent studies in higher-order harmonic generation (HHG)^23^ observe very large differences in the harmonic yield between the same pair of isomers. In the publication, they concluded (and later “softened” the conclusion^24^) that large differences in the strong-field ionization between molecular isomers is the underlying reason. On the contrary, Le *et al*. in ref. 25 present theoretical results, based on quantitative rescattering (QRS) theory, in which the large HHG yield ratio is explained by the interplay in the angle-dependence of both tunneling ionization and photorecombination. Nevertheless, in HHG, XUV radiation due to the motion of the electron in the continuum is driven by a strong laser field. The HHG process depends on ionization as a first step^26^,^27^,^28^,^29^, and thus provides a somewhat convoluted measurement of the ionization. Therefore, a direct measurement and comparison of the ionization yields is needed to shed some light on this controversy.

## Experimental Results

**Figure 1:**
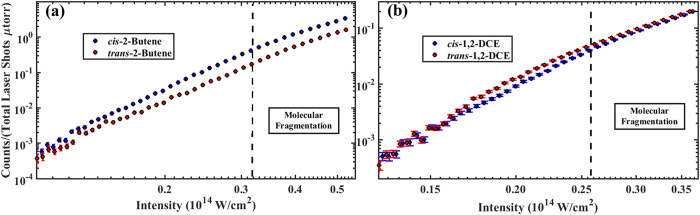
Ionization yields of the first ion of (**a**) 2-butene and (**b**) 1, 2-DCE (35–35 isotope) as a function of intensity (log-log).

The ionization yields as a function of intensity for each isomer pair is shown in Fig. 1. Experimental ionization yields were measured using an time-of-flight mass spectrometer (TOFMS). The intensity range for each isomer was bounded by two phenomena, statistical significance for low intensity and molecular fragmentation for high intensity. Each intensity data point per sample was determined by an average of *N* = 64000 laser shots (linearly polarized, 30 fs pulse duration, 790 nm center wavelength) and the statistical error was calculated as the standard error. Please see the **Methods: Experimental Setup** section for more details.

**Figure 2:**
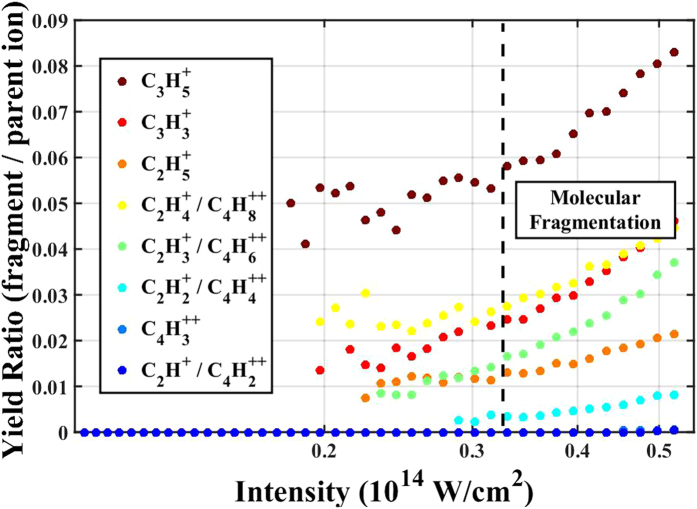
Ratio of the ionization yield of a molecular fragment and the parent ion of *cis*-2-butene. Molecular fragmentation is significant beyond 
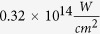
.

In each figure, the intensity range is extended to show the effects of molecular fragmentation on the ratio of the yields. Fragmentation analysis, shown in Fig. 2, compared the ratio of the fragment ion to the parent ion. Since there is always some nonzero probability that fragmentation will occur, we found that the rate of fragmentation changed at the same rate with respect to intensity as the rate for the parent ion until the saturation intensity was reached. This intensity is clearly determined by the change in slope of the yield ratio (fragment/parent ion) as seen in Fig. 2. Note that we only show the fragmentation analysis for *cis*-2-butene, however, we found similar trends for all isomers studied and determined saturation intensities appropriately.

**Figure 3:**
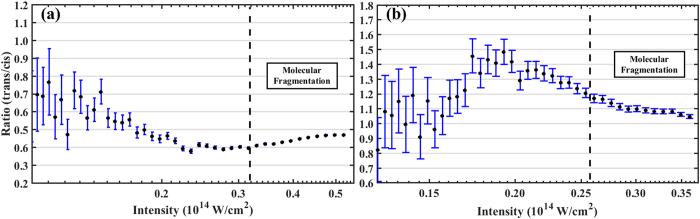
Ratio of the yield of the parent ion of (**a**) 2-butene and (**b**) 1, 2-DCE (35–35 isotope) as a function of intensity (semi-log).

For each pair of isomers (see Fig. 5(b)–(e)), we investigated the ratio of the yields (trans/cis) of the first ion of the parent molecule as a function of intensity. The ratios are depicted in Fig. 3(a) and (b). The ionization yields as a function of intensity for 1,2-DCE, Fig. 1(b), are statistically significant in the intensity range 0.12–
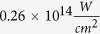
 before fragmentation. The ratio, Fig. 3(b), is roughly 1.0–1.4 within that intensity range. For this isomeric pair, the *trans*-isomer yield dominates the *cis*-isomer yield. The ratio changes such that the *cis*-isomer yield has a stronger presence at higher intensity compared to lower intensity, however, it never dominates the *trans*-isomer yield. Molecular fragmentation becomes visible at about 
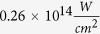
 where the *trans*-isomer fragments before the *cis*-isomer which is visible in the change in slope of the ratio to show a decrease in *trans*-isomer yield contribution. Note, the chlorine in 1, 2-DCE has two stable isotopes found in nature, Cl-35 and Cl-37 with 75.76% and 24.24% abundance^30^, respectively. This results in three different ionization peaks in the time-of-flight spectra. In this paper, we only show data for the 35-35 isotope pair.

The ionization yields as a function of intensity for 2-butene, Fig. 1(a), are statistically significant in the intensity range 0.10–
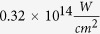
 before fragmentation. The ratio, Fig. 3(a), is roughly 0.4–0.7 within that intensity range. For this isomeric pair, the *cis*-isomer yield dominates the *trans*-isomer yield. The ratio changes such that the *cis*-isomer yield has a stronger presence at higher intensity compared to lower intensity. Molecular fragmentation becomes visible at about 
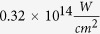
 where the *cis*-isomer fragments before the *trans*-isomer which is visible in the change in slope of the ratio to show a decrease in *cis*-isomer yield contribution.

By comparing the two ratios of the two pairs of stereoisomers, we observe that no isomeric structure (cis or trans) dominates compared to the other, therefore we do not observe a strong influence of molecular polarity on the molecular ionization. In addition, the trend of the ratios before fragmentation are not the same between the two pairs. For 1, 2-DCE, there is a positive slope in the ratio in favor of the non-polar *trans*-isomer. 2-butene, however, is the opposite with a negative slope in favor of the polar *cis*-isomer. In both cases, however, the dominating isomer is the first to fragment.

We note that we ignore the possibility of any dynamic effects of the molecular samples in the presence of the laser field. Our laser pulse duration was only 30 fs long (FWHM) and at low intensities, we do not expect significant alignment for these relatively heavy molecules. Similarly, we do not expect significant molecular structural rearrangement during this short time period. Further investigations are needed, however, in particular with different laser wavelengths, to rule out possible resonance effects to an excited state.

## Theoretical Results

To simulate the experimental measurement we utilize three different approaches: (i) strong-field approximation (SFA)^1^,^21^, (ii) molecular tunneling ionization theory (molecular orbital Ammosov-Delone-Krainov, MO-ADK)^16^, and molecular orbital Perelomov-Popov-Terent’ev (MO-PPT)^9^,^22^. Since *cis*-isomers are polar molecules, we also extended these calculations to include Stark corrections^31^,^32^,^33^.

**Figure 4:**
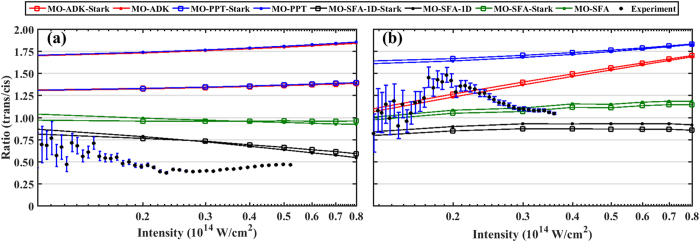
Ratio of the yield of the parent ion of (**a**) 2-butene and (**b**) 1, 2-DCE (35–35 isotope) as a function of intensity with theoretical and experimental data (semi-log). Scattered points are experimental results, connected scattered points are theoretical results. For an explanation of the different theoretical methods see the **Theoretical Results** section.

In our calculations we use ground-state electronic wavefunctions obtained from the Gaussian quantum chemistry code^34^ and employ the augmented correlation consistent polarized valence triple-zeta (aug-cc-pVTZ) basis set at the Hartree-Fock level. Within the single-active-electron (SAE) approximation, we take the highest occupied molecular orbital (HOMO) as the “ground state”. With these wavefunctions, the SFA calculation was then carried out for each target for different laser parameters integrating over all electron emission directions. The calculations were carried out within the clamped nuclei approximation in which the nuclei are fixed at their equilibrium positions of the neutral molecule (implying vertical ionization in the 1-D case). The geometry optimizations were performed and the results are consistent with other calculations (reported in NIST Webbook, for example). The results are shown in Fig. 4 for a typical range of the laser intensities. As one can see, the trans/cis intensity ratio for 1, 2-DCE is of order 1 (±50% or so) for both theory and experiment. Note that theoretical results from Wong *et al*.^23^ (see Erratum^24^) show somewhat smaller trans/cis ratios (close to 0.5), who used different intensities and/or wavelengths. The SFA trans/cis ratio for 2-butene is also close to 1, while experimental ratio is close to 0.5. Figure 4(a) also reveals that, the agreement between theory and experiments for trans/cis ratio for 2-butene is somewhat improved by considering only electron emission along polarization axis (MO-SFA-1D-Stark), although such trend is not quite clear for 1, 2-DCE.

We have also found that the intensity ratios are relatively stable with respect to the different basis sets and methods (Hatree-Fock and density functional (DFT) such as B3LYP) used in Gaussian. The dipoles of the HOMO can change up to 20% depending on the basis sets and methods used, however, the Stark correction within the SFA only slightly changes the ionization yield ratios (square points). Our calculations were done with the permanent dipoles of the active electron “ground-state” wavefunction (i.e. HOMO) of −0.97 au and −0.75 au for *cis*-2-butene and *cis*-1, 2-DCE, respectively. For the *trans*-isomers, the dipoles are zero. Note, we did not attempt to go beyond the Hartree-Fock and DFT for strong-field ionization. Most of the theoretical treatments for strong-field ionization have been, so far, limited to the SAE approximation. Going beyond the Hartree-Fock approximation would also mean going beyond the SAE. We note that, so far, only in a few rare cases electron correlation was suspected to contribute to the total ionization.

Within the MO-ADK theory, we first extracted the molecular structure coefficients *C*_*lm*_ using the HOMO’s obtained from the Gaussian quantum chemistry code. This is done by matching the HOMO with its asymptotic wavefunction^16^ at some large distance *r*_*e*_. As it is well-known, this procedure is not quite satisfactory even with large basis sets which includes diffuse functions, since Gaussian-type orbitals decrease too rapidly at large distances^35^. Therefore, the ionization rate for each isomer obtained from the MO-ADK still changes quite significantly with distance *r*_*e*_, even with large basis sets, such as, aug-cc-pVTZ and aug-cc-pVQZ. Nevertheless, we found that the trans/cis intensity ratio are relatively stable when *r*_*e*_ ≈ 10 to 15 au. Our results from MO-ADK for the trans/cis intensity ratio is shown in Fig. 4, which is in a relatively good agreement with the SFA results. Here we took *r*_*e*_ = 10 and *r*_*e*_ = 14 au for 2-butene and 1, 2-DCE, respectively. Note that, in general, the validity of the MO-ADK might be questionable at low intensities used in the experiments. We, therefore, also provide here MO-PPT results^22^ which is expected to have a much broader range of validity compared to MO-ADK. Overall, SFA results agree better with experiments than both MO-PPT and MO-ADK. The Stark correction does not change the results significantly in all cases.

It should be noted that in the recent papers by Tolstikhin and collaborators^36^,^37^, an adiabatic expansion in parabolic coordinates approach has been developed to describe ionization process from molecules, including polar molecules. Madsen *et al*.^35^ have also shown that quite accurate wavefunctions at asymptotic distances can be obtained from Gaussian with a well-designed basis sets. Unfortunately, both of these approaches are limited to simple molecules so far.

## Discussion

In this paper we report the ionization yield of two pairs of stereoisomers, *cis*-, *trans*-1, 2-DCE, and *cis*-, *trans*-2-butene, as a function of intensity. The ratio trans/cis as a function of intensity is also reported for each isomeric pair. From a strong-field ionization perspective, because of the similar ionization potentials between the pairs, we would expect very similar yields (yield ratio ≈1). Especially in this case where the molecules where randomly oriented. Therefore, it is surprising that our findings indicate that one isomer dominates its stereoisomer counterpart by a factor on the order of 1.5–2. We show that there is no single dominating configuration, *cis*- versus *trans*-isomer. Such lack of dominant configuration suggests that molecular polarity is not a major contributor to the rate at which a molecule ionizes under strong fields.

To provide a marker on the current status of theoretical molecular ionization models, the measured ionization ratios are compared to theoretically-calculated ionization yield ratios. We consider stark-corrected SFA to be one of the best methods available in terms of calculating molecular ionization rates. However, the experimental results do not match the SFA theory, even within the error of the measurement for all samples studied. Similarly, the MO-ADK and MO-PPT results are a poor fit as well. This is expected for MO-ADK because it is suited for the tunneling regime and our experiments are performed when *γ* > 1. MO-PPT, on the other hand, is better suited for a broader range of *γ*, however, the ratio has the least agreement with the experimental results. Although we can assign yield ratios confidence based on the model, the difference in ratio values between the different theoretical methods, SFA, MO-ADK, and MO-PPT, is large enough to question how well the theory can actually represent the experiment. It should be noted that typical, yet computationally costly corrections were neglected in the molecular calculations. One important of such omissions is focal volume averaging. Also, although many steps were taken to report only the ionization of parent molecular ions prior to fragmentation, the dynamics of such complex polyatomic molecules are not included in the theoretical calculations and might have a large influence on the ionization yield even in the intensity range of our experiment.

It should be pointed out that our results are in disagreement with previous studies^23^,^24^, where the difference in HHG yield is attributed to differences in ionization yields. The calculated ionization yield ratios were 5 for 2-butene and 2 for 1, 2-DCE. The *cis*-isomer dominates in both cases. The authors in refs 23,24 do, however, maintain that the angular distributions of the electron for 800 nm, 1300 nm and 1500 nm look qualitatively similar to each other. At 790 nm, we experimentally observed ionization differences that do not support the current explanation for the large yield differences present in the HHG studies. Meaning, it is likely direct ionization measurements in the near-infrared will yield similar results. Further studies at longer wavelengths are required.

## Methods

### Experimental Setup

**Figure 5:**
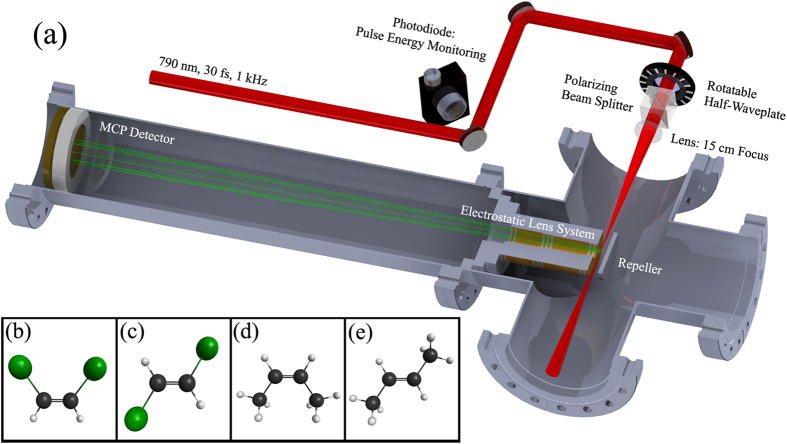
(**a**) Experimental optical setup and time-of-flight mass spectrometer. Geometries of the molecules: (**b**) *cis*- and (**c**) *trans*-1, 2-dichloroethylene, (**d**) *cis*- and (**e**) *trans*-2-butene.

See Fig. 5(a) for a layout of the experimental setup. Our experiment makes use of the High Intensity Tunable Source (HITS) laser, a Ti-Sapphire multipass, cryo-cooled, CPA amplified^38^ laser with 790 nm central wavelength, ≈30 fs FWHM pulse duration, 1 kHz rep. rate, and ≈50 nm bandwidth^39^. The intensity of the laser is controlled by a rotatable half waveplate and a polarizing beam cube. This guarantees that the light entering our chamber is always polarized linearly and perpendicular to the surface of our detector while keeping the mode profile constant. Near transform limited pulses in the interaction region are obtained by compensating for the group velocity dispersion (GVD) of the optical elements in the beam path using the grating pair in the amplifier compressor. The light (irised to about 1.5 cm from an original 1/*e*^2^ width of 2.2 cm) is focused into a time-of-flight mass spectrometer (TOFMS)^40^ with a f = 15 cm plano-convex lens. Each sample is introduced to the chamber via a gas line ending with a glass capillary with a hollow core diameter of 200 *μm*. The capillary is about 6 cm from the interaction region producing a homogeneous gas density throughout the interaction region. The background base pressure is ≈10^−9^ torr and for an experiment is brought up to a working pressure in the range of about 10^−7^ to 10^−6^ torr depending on the sample. The ions generated in the interaction region by the laser pulse are repelled toward a micro-channel plate (MCP) detector and converted into a time dependent voltage.

The ions, universally labeled by their mass and charge, are identified by their arrival time to the detector. Using a fast oscilloscope (model: picoscope 5203), we perform single-shot measurements of all of the ions produced. The energy of every laser pulse is measured in parallel with the ions. The oscilloscope obtains complete time-of-flight and photodiode traces at 1 kHz. This allows us access to not only the information of the parent ion, but also any other event that may have occurred with that particular laser pulse. Using a multichannel oscilloscope capable of recording data at the repetition rate of the laser allows for perfect synchronization between the energy and fragments measured. With the yield and energy measurements, we can “tag” ions to pulse energy. Such tagging permits the discrimination of events with specific values of peak intensity. An ionization event can then be placed in another intensity bin, or discarded entirely. Due to the highly non-linear nature of the process, intensity discrimination is crucial in comparing strong-field ionization effects as a function of intensity. We found that, on average, the laser energy fluctuated with a 2.15%–2.5% single-shot standard deviation over each experiment. We assign an energy to each data point via an angle-to-energy calibration associated with the *λ*/2-waveplate angles. The calibration was performed by measuring the power at a variety of angle positions of the waveplate within the full range of energies and finding the best fit based on those measurements. The fitted model for each experiment had a relative standard error for the forecast of <0.5% for all energies used.

The ionization yield is determined, post-experiment, by gating the signal around the expected time of arrival of a particular ion and either integrating the total signal or counting the individual hits on the detector for every laser shot. We found that at low intensity, before saturation, the counting method was best and is how the data is presented in this paper.

To account for experimental fluctuations, we monitor and correct for pressure variations, so that each yield is considered pressure (density) independent. For our experiments, which can have data acquisition times on the order of hours, we found that the pressure would drift over time. The pressure would reduce on average from the initial starting pressure by a factor of 0.5 for 2-butene, and 0.65 for 1, 2-DCE. The pressure measurement itself is not performed at a single-shot rate because the change in pressure is very slow compared to the repetition rate of the laser, therefore, we take a pressure reading every 400 laser shots. We use a Bayard-Alpert ionization gauge (BAG) and a Granville-Phillips 330 Ionization Gauge Controller with an accuracy of 3%. Although the calibration constants for the BAGs for the samples used in this paper are not available, for impact electrons in the range of 50–150 eV, we argue that for each isomeric pair, the cross section is nearly identical. Our assumption is based on reported studies of comparative cross sections for different molecular isomers in the same energy range^41^,^42^,^43^. Taking all these factors into consideration we assume that the pressure measurements for each pair are calibrated within 10% or less.

### Intensity Calibration

Intensity values reported here are determined through a calibration procedure utilizing the ionization yield of argon as a function of energy under the same experimental conditions as the isomer experiments run. This is a similar procedure as that used in ref. 29, but in a full gaussian volume, where the ion yield from a calibration gas as a function of pulse energy is fit to theory. Theoretical ionization yields for argon were determined using a method developed by Yudin and Ivanov^13^ termed nonadiabatic tunnel ionization theory (NTI). Yields are reported after volume averaging and cycle-averaging^44^,^45^,^46^. In this case, NTI ionization rates are superior to other commonly used theoretical methods, such as, the ADK (Ammosov-Delone-Krainov) model^47^ because the calibration is over an intensity range that covers both the multi-photon and tunneling regimes. We comment that we choose to use the NTI theory for its ease of implementation and acknowledge that there are other equally valid and proven methods available, such as, the original Perelomov-Popov-Terent’ev (PPT) theory^9^,^10^,^11^ and the improved PPT model^12^ as shown in ref. 48.

For the theoretical calculations we follow ref. 44 and the equation for the focal volume of a gaussian beam was found in ref. 45. Again, the difference is that we use the NTI rates. The field envelope was determined experimentally using the FROG technique^49^ with a reconstruction error of 0.3% (reconstruction software: FROG 3.2.2 by Femtosoft Technologies 2006). The spatial mode was assumed a gaussian and parameters, such as spot size, become part of the intensity calibration constant described in the next paragraph^44^.

**Figure 6:**
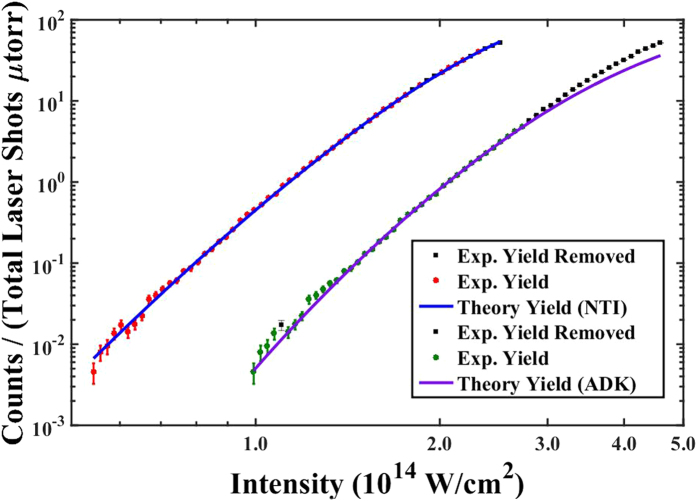
The experimental ionization yields as a function of intensity are fit to cycle-averaged, volume averaged, theoretical ionization yield curves generated using NTI and ADK theory. The corresponding calibration constants are *α* = 4.5 × 10^12^*W*/*cm*^2^*J* with 8 points removed and *α* = 8.25 × 10^12^*W*/*cm*^2^*J* with 20 points removed, respectively.

The theoretical model produces the yield as a function of peak laser intensity, *I*_0_, *N*_*thry*_(*I*_0_), which is then fit to the experimentally measured yield as a function of pulse energy, *E*_*exp*_, *N*_*exp*_(*E*_*exp*_). Since we care only about relative yields, and are interested in calibrating our energy into peak intensity, the actual fitting is done between the functions *N*_*exp*_(*αE*_*exp*_) and *cN*_*thry*_, where *α* is the energy-intensity calibration constant and *c* is a normalization parameter for the theoretical yield. The fitting for Ar^+^ using the NTI model is shown in Fig. 6. Details of the fitting procedure, which involve a weighted-exponential least-trimmed sum resistant regression method^50^ are given in the Supplementary Information section.

## Additional Information

**How to cite this article:** Zigo, S. *et al*. Ionization Study of Isomeric Molecules in Strong-field Laser Pulses. *Sci. Rep.* **7**, 42149; doi: 10.1038/srep42149 (2017).

**Publisher's note:** Springer Nature remains neutral with regard to jurisdictional claims in published maps and institutional affiliations.

## Supplementary Material

Supplementary Information
