# Supplementary material for: Ionization Study of Isomeric Molecules in Strong-field Laser Pulses
Source: Sci Rep. 2017 Feb 10;7:42149. doi: 10.1038/srep42149 (PMC5301495; doi:10.1038/srep42149)
Supplement: Supplementary Information [file srep42149-s1.pdf]

Ionization Study of Isomeric Molecules in Strong-field Laser Pulses

S. Zigo, A. T. Le, P. Timilsina, and C. Trallero-Herrero

J.R. Macdonald Laboratory, Department of Physics, Kansas State University, Manhattan, Kansas 66506, USA

ABSTRACT

Through the use of the technique of time-of-flight mass spectroscopy, we obtain strong-field ionization yields for randomly oriented 1,2-dichloroethylene (1,2-DCE) ($C_2H_2Cl_2$) and 2-butene (C_4H_8). We are interested in studying the effect of conformal structure in strong-field ionization and in particular the role of molecular polarity. That is, we can perform strong-field ionization studies in polar vs non-polar molecules that have the same chemical composition. We report our findings through the ionization yields and the ratio (trans/cis) of each stereoisomer pair as a function of intensity.

Supplementary Information

Intensity Calibration Method: Robust Fitting

As mentioned in the main text, theoretical calculations follow[1] and the equation for the focal volume of a gaussian beam was found in[2,3]. The fitting is done between the functions $N_{exp}(\alpha E_{exp})$ and cN_{thry}. The experimental data set $N_{exp}(\alpha E_{exp})$ is the yield of Ar^+ as a function of 58 intensity points. Since our ionization yield is exponential in nature and the experimental error of the yield is inhomogeneous with greater statistical significance at higher intensity, we utilize the method of weighted-exponential least squares fitting to find the correct intensity calibration factor, α.

Even while utilizing some of the most modern theories for the ionization of atoms, it is still challenging to incorporate all possible experimental parameters into a theoretical model. For example, although volume averaging is used to simulate a real experimental effect, the precise spatial distribution of the laser through the focus is very hard to measure accurately. In order to make our intensity calibration method more robust, we combine the weighted-exponential least squares with a resistant regression method termed as the least trimmed sum (LTS) of squares method. In this method, after a fit is found, the weighted squared residuals are sorted from smallest to largest and a predetermined number of data points with the largest residuals are removed. The remaining data points are then used to determine a new fit. The process is repeated until a "stable" intensity calibration factor is found. It is important to note that this is an iterative procedure and the data points removed can change on every iteration. For more information on robust fitting methods, see[4].

In our analysis, we test two different ionization models (ADK and NTI) to determine the correct intensity calibration factor, α, $I = \alpha E$. In our experiment, for argon, we are in both the multi-photon and tunneling ionization regimes with $\gamma \approx 1$. We intentionally chose ADK as an example of a poor ionization model in this particular regime[5] to demonstrate that our proposed intensity calibration analysis gives insight into the validity of a chosen ionization model. NTI, on the other hand, is valid for any γ[6] and is an example of how the intensity calibration analysis sheds light on additional deviations between the experimental value and the theoretical parameters chosen to describe the experiment. It should be noted that a reliable α can only be identified when a "good fit" between experiment and theory is achieved. When performing regression analysis, there are four main conditions that must be satisfied before a fit is considered good: linearity, statistical independence, normality, and homoscedasticity. The only condition that is not automatically met in our intensity calibration procedure is homoscedasticity, a constant variance of the residuals as a function of intensity.

For each model, we analyze the properties of the unique residual sets as a function of data points removed. Such properties include standard deviation of the weighted residuals, Fig. 1(a), average value of the weighted residuals, Fig. 1(b), and retrieved intensity calibration coefficient, Fig. 1(c). In addition to these properties, we visualize a map of which points were removed during the fitting procedure in order to give insight into whether or not the ionization models are valid for the conditions of the experiment, see Fig. 2(a) and Fig. 2(b).

Results: ADK Method A usable intensity calibration factor, α, was not achieved with this model. In Fig. 1(a), the standard deviation of the weighted residuals does not fall below 2 until after 20 points are removed and never reaches a "steady-state". Similarly, the average values, Fig. 1(b), of the residuals never reach a "steady-state", either. This means that the removed points are having large effects on the fit and it is unlikely that homoscedasticity was ever reached.

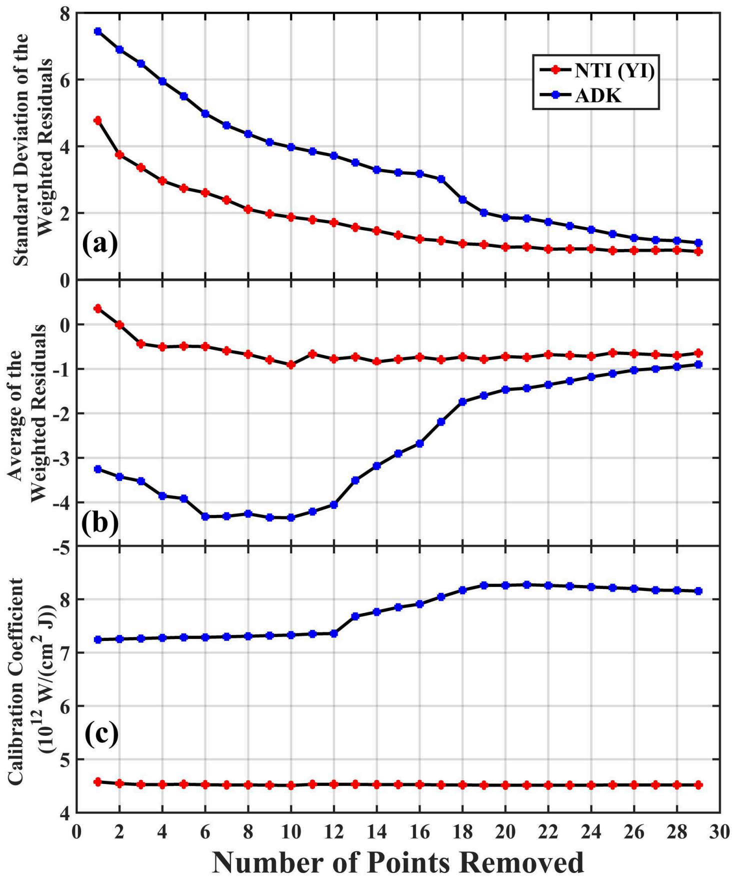

Figure 1. The (a) standard deviation of the residuals, (b) average of the residuals, and (c) retrieved intensity calibration coefficients as a function of the number of points removed in the robust fitting procedure using both NTI and ADK ionization methods.

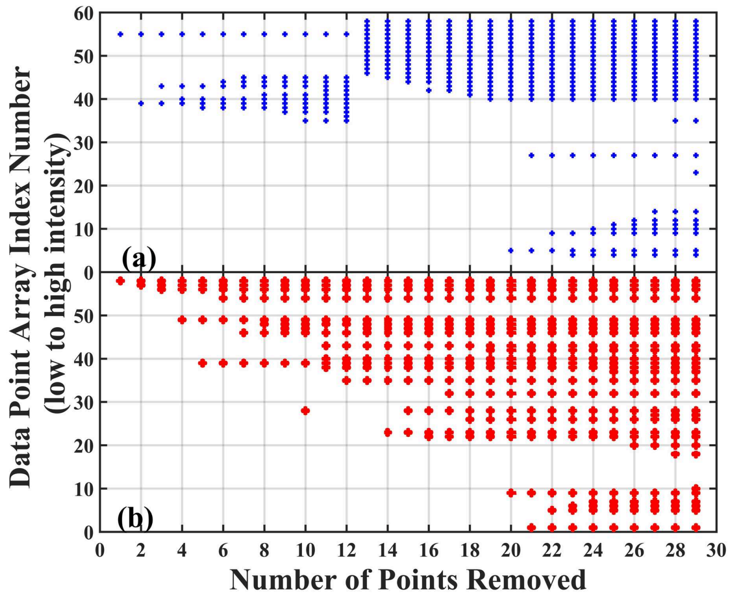

Figure 2. A map of which points in the data set were removed as a function of the number of points removed in the robust fitting procedure using (a) ADK theory and (b) NTI theory.

Additionally, notice how in Fig. 2(a), the removed points are grouped together in a particular intensity range, and then, there is a drastic change after 12 points are removed. The removed points remain grouped together, but consist of a different set of data points in a different intensity range. Note, this drastic shift is also reflected in the intensity calibration coefficients shown in Fig. 1(c), where an increase of 13% occurs from 12 to 18 data points removed. A visualization of the experimental data and fitted theoretical data is shown in Fig. 6 of the main text for ADK with 20 points removed.

It is enough to condemn these fits based on the high standard deviation values of the weighted residuals and the non-constant average values, however, that does not determine the mechanism at fault. In Fig. 2(a), the first 12 removed data points of the ADK method are removed from the middle of the intensity range. Past 12, suddenly, removed data points come from the high intensity regime. The fact that the removed points originally came from the middle intensity regime indicates heteroscedasticity caused by a poor model. It is unlikely that the middle intensity regime would be at fault more than the low intensity regime in terms of a carefully performed experiment. It is clear that the middle residuals were being compensated by both the high and low intensity regimes. Once that was no longer the case, i.e. enough points were removed, the model switched to a different fit that favored the middle intensity regime more. It should be noted that the high intensity regime was anticipated to be favored based on the knowledge that ADK works better in the tunneling regime, $\gamma << 1$. In practice, however, the lower intensity regime was favored. This is due to the small data set and larger experimental error in the low intensity regime which allows more flexibility in the fitting in that regime. One main disadvantage of the robust fitting analysis is that sufficient data points over a large enough intensity range need to be taken in the experiment in order to determine a "good fit". Misleading results can be observed if too many points are removed. The absolute break down of the method is when more than half of the points are removed.

Results: NTI Theory In this example, we identify a "good fit" and reliable calibration within the error of both the experiment and theoretical model. The argon experimental ionization yield data set is identical to the one used in the previous subsection where ADK theory was utilized. In Fig. 1(b), we reach a "steady-state" average value of ≈ -0.75 after the removal of 8 data points. Unfortunately, a "steady-state" alone does not indicate that the fits are now homoscedastic. In Fig. 1(a), a standard deviation of 2 or lower occurs after 8 data points are removed. Note that in order to confirm homoscedasticity, the variance of the residuals must be constant even if more points are removed from the data set. Unfortunately, the small data set prevents a constant standard deviation. However, we can infer homoscedasticity based on a "steady-state" of the average residual value, meaning the remaining "would be removed" data points do not significantly contribute to the overall fit and, also, the remaining "would be removed" data points become spread out over the entire data set as shown in Fig. 2(b). Note, the point at which homoscedasticity is reached is also reflected in the values of the intensity calibration coefficients, however, much more subtly. In Fig. 1(c), the coefficients do change as a function of data points removed, however, the change from all points to "steady-state" is small and on the order of 1%. The coefficient used for this article was $\alpha = 4.5 \times 10^{12}$ resulting from the removal of 8 data points. For a visualization of the experimental data and fitted theoretical data, see Fig. 6 in the main body.

The main advantage of the robust fitting procedure and its inherent analysis is the insight it gives into the quality of the experiment. In Fig. 2(b), the main data points contributing to the misidentification of the true intensity calibration coefficient were data points in the high intensity regime. In this regime, two things occur, detection saturation and yield saturation with volume averaging effects. Although both issues could be contributing, it is more likely that detection saturation is occurring. In the counting method, the yield analysis described in the main article, there is the possibility of being unable to temporally resolve single counts if too many arrive to the detector at similar times per laser shot. This causes an artificial decrease in yield at high intensity which would not be expected to be reflected in any of the ionization models. A volume averaging effect, on the other hand, should be less likely to contribute since the ionization model has already taken that into account. In our intensity calibration model, we do assume a gaussian laser profile. If this is not the case in the actual experiment, then volume yield saturation may influence the ionization yields significantly starting at the incorrect intensity. This would cause the model to also be incorrect at higher intensities.

References

1. Chang, B., Bolton, P. R. & Fittinghoff, D. N. Closed-form solutions for the production of ions in the collisionless ionization of gases by intense lasers. *Phys. Rev. A* **47**, 4193–4203 (1993). URL `http://link.aps.org/doi/10.1103/PhysRevA.47.4193`.

2. Gibson, G. N., Freeman, R. R., McIlrath, T. J. & Muller, H. G. Excitation and ionization dynamics in short-pulse multiphoton ionization. *Phys. Rev. A* **49**, 3870–3874 (1994). URL `http://link.aps.org/doi/10.1103/PhysRevA.49.3870`.

3. Augst, S., Meyerhofer, D. D., Strickland, D. & Chin, S. L. Laser ionization of noble gases by coulomb-barrier suppression. *J. Opt. Soc. Am. B* **8**, 858–867 (1991). URL `http://josab.osa.org/abstract.cfm?URI=josab-8-4-858`.

4. Kutner, M. H., Nachtsheim, C. & Neter, J. *Applied Linear Regression Models* (McGraw-Hill/Irwin, 2004), 4 edn.

5. Zhao, S.-F., Le, A.-T., Jin, C., Wang, X. & Lin, C. D. Analytical model for calibrating laser intensity in strong-field-ionization experiments. *Phys. Rev. A* **93**, 023413 (2016). URL http://link.aps.org/doi/10.1103/PhysRevA.93.023413.

6. Yudin, G. L. & Ivanov, M. Y. Nonadiabatic tunnel ionization: Looking inside a laser cycle. *Phys. Rev. A* **64**, 013409 (2001). URL http://link.aps.org/doi/10.1103/PhysRevA.64.013409.
